# The patient safety culture perception of Turkish nurses who work in operating room and intensive care unit

**DOI:** 10.12669/pjms.332.11727

**Published:** 2017

**Authors:** Selda Rizalar, Sacide Yildizeli Topcu

**Affiliations:** 1 Selda Rizalar, PhD, RN. School of Health Science, Istanbul Medipol University, Istanbul, Turkey; 2 Sacide Yildizeli Topcu, PhD, RN. Faculty of of Health Science, Trakya University, Edirne, Turkey

**Keywords:** Critical care, Nurses, Operating Rooms, Patient Safety, Perception

## Abstract

**Objective:**

To determine the patient safety culture perception of operating room and intensive care nurses and the factors affecting this perception.

**Methods:**

This descriptive study was conducted on 232 nurses working in a Turkish city hospitals. The data obtained from the nurses were collected using personal information form and Patient Safety Culture Scale (PSCS) from June to July 2015.

**Results:**

The total score average of the nurses on the PSCS was 2.58±0.39. The nurses obtained the highest score on the employee behavior subscale, and the lowest score on the the adverse event reporting system subscale. No significant difference was found between the total score averages of the PSCS of the operating room and intensive care nurses (p>0.05).

**Conclusion:**

The patient safety culture score average of the operating room and intensive care nurses was at medium level. In addition, being able to choose the unit in which they worked, working day or night shifts, and being educated on patient safety were found to affect the patients safety cultures of the nurses (P<0.05).

## INTRODUCTION

Patient safety is defined as preventing patients from being harmed. This includes all measures taken by health institutions and their personnel in order to prevent harm done to patients by healthcare services.^1^,^2^ The basics of developing a safety culture in healthcare are to decrease errors and improve the total quality of healthcare.^3^-^5^

The perception of the importance of a safety culture may vary among units of a hospital.^3^ Patient safety is crucial in ICUs. The patients in the Intensive Care Unit (ICU) have a higher risk of being harmed by medical errors and the lack of a positive patient safety culture.^6^,^7^ The observers in a study on ICU units reported that 17% of the patients in these units were reported to have serious side effects.^6^,^7^ There is evidence that medical errors and unexpected events increase in proportion to the severity of the disease, and to the intensity and complexity of the care that is provided in the complex and high-stress environments of the ICU.^8^ Operating rooms(OR) also have characteristics similar to those of intensive care units and can be even more risky in terms of patient safety. Therefore, the personnel working in these units are expected to be more skilled in patient care and safety.^6^-^8^

Communication and cooperation should be established between healthcare personnel and managers to develop a positive patient safety culture.^9^ It is very important that hospital leadership focuses on and values patient safety in order to create and maintain a positive patient safety culture. The perception and attitudes of all personnel regarding patient safety should be measured in order to create and improve the patient safety culture.^3^,^6^,^10^ The results of these measurements provide useful information to managers and leaders in determining the concerns of patients, and the areas that need to improve the quality of care.

Nurses have an important role in ensuring and maintaining patient safety because they provide direct care to patients. They are responsible for protecting the patients from the possible dangers and preventing the unwanted results of initiatives in each environment in which they work.^2^ Nurses play a key role in improving the quality of healthcare by means of patient safety initiatives. Therefore, determining the perceptions of nurses regarding patient safety culture and the factors affecting patient safety through the use of the measurement tools will form a basis to create and develop a positive safety culture.

## METHODS

This descriptive and cross-sectional study was conducted on 232 nurses working in the ICUs and ORs in a city in Northern Turkey between 1^st^ and 30^th^ June, 2015. There are three hospitals in this city and wich are (university hospital, research hospital, and state hospital) These hospitals were accredited by ISO and/or JCI. The nurses who volunteered to participate in the study were included. Sampling selection was not applied; it was aimed to reach all personnel. The total number of nurses working in the operating rooms and intensive care units of hospitals was 355. The data obtained from the 232 nurses who filled out the questionnaire were used in this study (response rate was 65%, participant number and total number of nurses were 232 and 355, respectively.)

### Data collection

The data obtained from the nurses were collected using personal information Form included eight items about personal and professional features and “Patient Safety Culture Scale” developed in Turkish by Turkmen *et al*.^11^ The Patient Safety Culture Scale includes 51 items and consists of five subscales that include Management and Leadership (17 items), Employee Behavior (14 items), Adverse Event Reporting System (5 items), Staff Education (7 items), and Care Environment and Technology (8 items).

Each item is answered on a 4-point Likert scale from 1(completely disagree) to 4(completely agree). Scores approaching 4 show a positive attitude toward patient safety culture, and scores approaching 1 show a negative attitude toward patient safety culture. In the original study on the reliability and validity of the scale, Cronbach’s α coefficients were 0.97 for the total scale score and between 0.83 and 0.92 for the subscales.^11^

### Procedure

The questionnaire Forms were provided by the researchers in the services after making the necessary explanation to the patients on the study. The nurses were given time to fill out the Forms and then they were collected. The questionnaire Forms were answered by 232 of the nurses working in the said clinics of the hospitals. Some nurses were on (sick) leave between the dates of this study and some nurses did not want to participate in the study. This study was conducted between June 1 and 30, 2015.

### Ethical Considerations

The study was conducted after formal permissions for the study were obtained from the Directorates of the Hospitals and the Ethic Commission of university hospital (IRB file no: OMU-KAEK 2015-174). Before the launch of the research, nurses were informed about the subject and the objectives of the research. Personal information would remain confidential and would only be used for the research data. Verbal and written permission were obtained from the nurses who volunteered to participate in the research.

### Data analysis

The data obtained from the study were evaluated in SPSS 16.0 program(SPSS Inc, ChicagoIl, USA). The frequencies and percentages of nurses were used to analyze their sociodemographic characteristics during data analysis. The score averages and standard deviation were used in calculating the nurses’ PSCS scores. The t test and analysis of variance were used in comparing the nurses’ PSCS scores according to their sociodemographic characteristics. P<0.05 was considered statistically significant for all tests.

## RESULTS

In this descriptive study in which 232 nurses participated, it was found that the average age of the nurses was 32.68 years (SD=5.77), 70.3% had a B.S.degree, 89.9% were working as bedside staff nurses, 82.8% were educated on patient safety, 39.5% had professional experience of ten years or more, 39.2% were working in operating rooms, and 60.8% were working in intensive care units. The nurses working in each of these three hospitals were working 40 hours per week. Some nurses always worked during the day(25%), some on worked the night shift sometimes(37.1%), and some always worked the night shift (37.9%).

The total score average of the nurses on the patient safety culture scale was 2.58±0.39, and the subscale score averages were 2.56±0.45 for management and leadership, 2.64±0.56 for staff education, 2.44±0.56 for adverse event reporting system, 2.61±0.49 for care environment and technology, and 2.65±0.46 for employee behavior. The nurses obtained the highest score on the employee behavior subscale, and the lowest score on the the adverse event reporting system subscale (Table-I). In the OR nurses, the total score average was 2.60±0.41, and the subscale score averages were 2.57±0.42 for management and leadership, 2.62±0.55 for staff education, 2.44±0.55 for adverse event reporting system, 2.64±0.52 for care environment and technology, and 2.71±0.47 for employee behavior. In the ICU nurses, total score average was 2.57±0.38 and the subscale averages were 2.55±0.46 for management and leadership, 2.66±0.57 for staff education, 2.44±0.56 for adverse event reporting system, 2.59±0.46 for care environment and technology, and 2.61±0.44 for employee behavior. Although no statistical difference was found, it was determined that the OR nurses obtained the highest score on the employee behavior subscale, while ICU nurses obtained the highest score on the staff education subscale(Table-I).

**Table-I:** Nurses’ Scores of the Patient Safety Culture Scale (N=232).

*Subscales*						

	*Management And Leadership*	*Staff Education*	*Adverse Event Reporting System*	*Care Environment and Technology*	*Employee Behavior*	*Total Score of The Scale*

Scores	M +SD	M +SD	M +SD	M +SD	M +SD	M +SD
All nurses	2.56±0.45	2.64±0.56	2.44±0.56	2.61±0.49	2.65±0.46	2.58±0.39
OR nurses(N=91)	2.57±0.42	2.62±0.55	2.44±0.55	2.64±0.52	2.71±0.47	2.60±0.41
ICU Nurses(N=141)	2.55±0.46	2.66±0.57	2.44±0.56	2.59±0.46	2.61±0.44	2.57±0.38
t test	0.260	-0.458	0.099	0.750	1.626	0.518
p value	0.795	0.647	0.921	0.453	0.105	0.605

A significant difference was found in the nurses’ score averages of the PSCS who chose to work on their units, compared to those who did not choose their unit. The subscale scores of the nurses who chose their unit were higher on the management and leadership and staff education subscales. A significant difference was found between the total score averages of the PSCS and score averages of the management and leadership, care environment and technology and employee behavior subscales in terms of the shift on which the nurses worked. The PSCS and subscale scores of the nurses who always worked in the night shift were lower than those who worked days and who sometimes worked the night shift. A significant difference was found between the score averages of management and leadership, staff education and employee behavior subscales in terms of being educated on patient safety. The total scores of those who received education were higher than those who did not receive education(Table-II).

**Table-II:** The Factors Affecting Patient Safety Cultures of the Nurses.

		*OR Nurses*						*ICU Nurses*					
		*Management and Leadership*	*Staff Education*	*Adverse Event Reporting System*	*Care Environment and Technology*	*Employee Behavior*	*Total score of PSCS*	*Management and Leadership*	*Staff Education*	*Adverse Event Reporting System*	*Care Environment and Technology*	*Employee Behavior*	*Total score of PSCS*
		*M+SD*	*M+SD*	*M+SD*	*M+SD*	*M+SD*	*M+SD*	*M+SD*	*M+SD*	*M+SD*	*M+SD*	*M+SD*	*M+SD*
The person who preferred unit	Herself/himself	2.67±0.34	2.74±0.46	2.46±0.40	2.70±0.47	2.78±0.43	2.67±0.33	2.58±0.52	2.78±0.54	2.42±0.56	2.62±0.46	2.60±0.42	2.60±0.39
	Administrator	2.42±0.48	2.46±0.63	2.41±0.72	2.56±0.59	2,62±0.52	2.49±0.48	2.53±0.42	2.56±0.58	2.45±0.56	2.57±0.47	2.62±0.46	2.55±0.38
	*t test*	2.726	2.299	0.395	1.257	1.637	1.934	0.636	2.204	-0.267	0.664	-0.140	0.845
	*p value*	0.008	0.025	0.695	0.212	0.105	0.058	0.526	0.029	0.790	0.508	0.889	0.400
Shift	Day shift	2.62±0.40	2.63±0.40	2.45±0.50	2.63±0.57	2.70±0.56	2.61±0.41	2.62±0.49	2.67±0.60	2.36±0.61	2.62±0.53	2.67±0.37	2.59±0.44
	Night shift	2.36±0.50	44±0.71	2.31±0.68	2.39±0.52	2.51±0.49	2.40±0.49	2.49±0.51	2.61±0.59	2.37±0.59	2.53±0.42	2.55±0.42	2.51±0.39
	Day and night shift	2.64±0.37	2.71±0.54	2.51±0.52	2.78±0.45	2.83±0.36	2.69±0.33	2.61±0.36	2.71±0.53	2.58±0.46	2.66±0.49	2.67±0.51	2.65±0.35
	*F*	3.386	1.692	0.869	4.017	3.195	3.609	1.290	0.462	2.397	1.099	1.193	1.793
	*p value*	0.038	0.190	0.423	0.021	0.046	0.031	0.279	0.631	0.095	0.336	0.306	0.170
Patient safety education	Not Received	2.03±0.34	1.95±0.42	1.97±0.54	2.21±0,47	2.29±0.31	2.09±0.28	2.44±0.37	2.38±0.58	2.44±0.58	2.59±0.46	2.48±0.45	2.47±0.38
	Received	2.63±0.39	2.69±0.51	2.50±0.53	2.69±0,51	2.76±0.47	2.65±0.38	2.59±0.48	2.73±0.55	2.43±0.56	2.59±0.47	2.65±0.44	2.60±0.38
	*t test*	-4.381	-4.169	-2.757	-2.655	-2.910	-4.214	-1.564	-3.096	0.084	-0.054	-1.822	-1.679
	*p value*	0.000	0.000	0.007	0.009	0.005	0.000	0.120	0.002	0.933	0.957	0.071	0.095

## DISCUSSION

The safety culture of the nurses who were working in pediatrics, psychiatry, and rehabilitation was found to be more positive than those who were working in emergency and operating rooms.^12^ Balanuye found that the patients were affected by the errors made by surgery clinic hospitals due to their heavy workload, and that this heavy workload affected the patient safety negatively.^13^ In this study, it was found that the patient safety culture scores of the operating room and intensive care unit nurses were at a medium level and no statistical significant difference was found between nurses who was working ICU and OR. The nurses obtained the highest score on the the employee behavior subscale and the lowest score on the care environment and technology and adverse event reporting system subscales. Also, patients safety cultures of the OR and ICU nurses was medium level and OR nurses obtained the highest score on the employee behavior subscale, while ICU nurses obtained the highest score on the staff education subscale. The PSC score averages were also found to be at the similar level in the study of Türkmen *et al*. and the study of Karaca & Arslan of nurses working in a private hospital using the same scale.^14^,^15^ A study conducted on 302 nurses working in the university hospitals in Iran, and a study conducted on 463 nurses in China indicated that patient safety perceptions of nurses weren’t at the desired level.^4^,^5^

In two studies conducted in the ICU by Huang et al., it was stated that PSC varied across ICUs and safety culture scores were mostly low to moderate, and nurses had lower safety culture scores than physicians.^16^,^17^ Göz & Kayahan found out that the patient safety score average of the nurses working in ICUs was lower than that of the nurses working in surgical units.^18^ In a study conducted in operating room and post-anesthesia care unit (PACU), Kaafarni et al. reported that PCS was similar or slightly worse than other hospital work areas and stated that this situation could be related to the complexity of tasks performed by the OR and PACU.^19^

In this study, both OR and also ICU nurses obtained the lowest scores on the Adverse Event Reporting System subscale. Gündoğdu & Bahçecik indicated that 72% of the nurses working in training and research hospitals and 73.5% of the nurses working in private hospitals didn’t submit a case report.^20^ Also, Karaca and Arslan reported similar findings.^15^ Dursun et al. stated in their study that 71.3% of the participants did not report any events that could pose a danger for patient safety.^21^ In a study on 74 OR nurses in a state hospital in Spain, PSC perception of the nurses was found to be positive, but some issues were found to be weak and 79.7% of the nurses didn’t submit case reports. In this study, the nurses believed that a positive PSC required personnel and the support of hospital management.^22^ Turkmen *et al*. found that the unit where the nurses were working in is related to the adverse event reporting system subscale.^14^ Other studies expressed the reason for not reporting the errors due to the fear of punishment, being accused, being blamed, losing prestige, and thinking of getting into trouble.^5^ The high rate of not reporting suggests that the nurses avoid submitting reports for fear of being punished.^14^

This study also aimed to determine the factors that affect the level of PSC. The shift on which they worked (days or nights) and receiving patient safety education were found to affect the PSCS total score of the OR nurses. A significant difference was found out regarding the fact that score average of the PSCS was higher in the OR nurses who received patient safety education and it was lower in the nurses who worked always at night shifts. The scores of management and leadership and staff education subscales were higher in the OR nurses who chose their unit. A significant difference was found between the score averages of the management and leadership, care environment and technology and employee behavior subscales in terms of whether the OR nurses worked days or nights. The subscale scores of the OR nurses who were always working at night were lower. It was stated in the literature that the units such as OR, ICU and emergency room were most complex health care settings in terms of the components influencing performance and PSC of the health care workers.^23^ Also, in a study it was pointed out that the patient outcomes were negatively affected and quality of the care was detoriated due to the fatigue and stress the nurses experienced.^24^ The literature supports the finding, which indicates that PSC was lower in OR nurses who were always working at night and the results of all studies represent that complexity of the work area and longtime and exhausting work periods of the nurses negatively affect patient safety and health care quality.

In this study it was found that, there was a significant difference between the PSC total score average in the OR nurses who received patient safety education and the score average of the staff education subscales in the ICU nurses who received patient safety education. The total scores and subscale score averages of the nurses who received education were higher than those who did not. Türkmen et al. found the subscale scores of the nurses who received patient safety being higher on the education on the management and leadership, employee behavior, staff education and adverse event reporting system subscales than those who did not receive patient safety education.^14^ Also, Haynes et al. reported that in their study conducted with OR workers such as nurses, surgeons and anaesthesia professionals, it was reported that the education and implementation of the WHO Safe Surgery Saves Lives-checklist increased the patient safety attitudes of operating room personnel.^25^ The report of the WHO emphasizes that patient safety is the responsibility of the whole institution, and in order to ensure the quality and safety, evidence-based practices should be developed and spread in the institutions and education and implementation of good practices should be improved.^3^ Results of the studies showed that PCS of the nurses can be affected by various factors such as characteristics of employees and units, working day or night shifts, workload and nurses’ fatigue and stress, administrative factors in units, and structures and properties of the hospitals.^24^

## CONCLUSION

The patient safety cultures of the nurses in this study were found to be at a medium level. In addition, being able to choose the unit in which they worked, working day or night shifts, and being educated on patient safety were found to affect the patients safety cultures of the nurses. These results demonstrate the importance of patient safety implementations and safety cultures of the employees in the units such as ICU and OR that have more risks related to the patients’ safety. In order to provide quality and safety patients care in this units, it is suggested that the institutions should be appropriately equipped as per quality management, continuously improve the content and the frequency of education. Additionaly, the conditions of the nurses who always work at the night shift should be evaluated and organized according to the individual preferences as much as possible.

### Author’s Contribution

Both authors of this paper have equally contributed to this study and approved the final version to be published.
